# Supplementary material for: Analyzing the collaborative development needs of grassroots centers for disease control and prevention using the Kano model: A case study of China’s Chengdu–Chongqing Economic Circle
Source: PLoS One. 2026 Apr 20;21(4):e0347594. doi: 10.1371/journal.pone.0347594 (PMC13094998; doi:10.1371/journal.pone.0347594)
Supplement: S1 File — (ZIP) [file pone.0347594.s001.zip › Supporting Information - Data/Delphi questionnaire.docx]

**Expert Consultation Letter on indicators of collaborative development needs of grassroots centers for disease control and prevention in Chengdu-Chongqing Twin City Economic Circle**

Dear Expert:

We are currently conducting the " Research on the index of collaborative development needs of grassroots centers for disease control and prevention(CDCs) in Chengdu-Chongqing Twin City Economic Circle." Through preliminary literature review, we have preliminarily established a draft evaluation indicator index for the collaborative development of grassroots CDCs. Given your academic achievements and influence in this field,we sincerely invite you to serve as a consulting expert for this project.The consultation results will be used solely for academic research purposes, and your relevant materials and responses will be strictly confidential. We hope you will accept and support this initiative. Thank you for taking the time to participate in this consultation despite your busy schedule. Your feedback will serve as a crucial basis for establishing the evaluation system.

This consultation consists of three components:①Evaluation Indicator Consultation Form, ②Expert Profile Form. Due to time constraints,please submit your feedback to the research team by March 1st. We sincerely appreciate your support and guidance!

For inquiries,please contact:

Zhang Min Telephone:13896028505 Email:75382237@qq.com

Correspondence Address:No.121，West Dushihuyuan Road,Shapingba District,Chongqing

Thank you again for your support. Wishing you success in your work and happiness in your life！

Research Group on indicators of collaborative development needs of grassroots centers for disease control and prevention in Chengdu-Chongqing Twin City Economic Circle

January 13, 2025

**Part One**

**Consultation Form for the Indicator of Collaborative Development of grassroots CDCs**

[Instructions]Please rate each indicator's importance using the 5-point Likert scale (Likert 5-scale),with 5 points for' very important',4 for' important ',3 for' moderately important ',2 for' not very important',and 1 for' unimportant'.If you find any statement inaccurate,please provide modifications or explanations in the remarks section.For additional content, please add it in the supplementary section below.

Table 1 Primary Indicator Consultation Form

| primary indicator | Importance score (1~5) | remarks |
| --- | --- | --- |
| Ⅰ Organizational Mechanism and Management |  |  |
| II Emergency Response |  |  |
| Ⅲ ability building |  |  |
| IV Information sharing |  |  |
| V Public Health Governance |  |  |
| VI Talent Training and Scientific and Technological Innovation |  |  |
| What additional content do you think should be added or supplemented? Please rate their importance. | | |

Table 2 Secondary Indicator Consultation Form

| primary indicator | secondary indicator | | Importance score (1~5) | remarks |
| --- | --- | --- | --- | --- |
| Ⅰ Organizational Mechanism and Management | 1-1 Joint Construction of Party Building Brands | |  |  |
|  | 1-2 Performance-based compensation(two options allowed) | |  |  |
|  | 1-3 Joint Construction and Sharing of Business Archives (Emergency Response Plans,Technical Solutions) | |  |  |
| What additional content do you think should be added or supplemented? Please rate their importance. | | | | |
| II Emergency Response | 2-1 Public Health Emergency Response Mechanism | |  |  |
|  | 2-2 Cross-regional case surveillance, investigation, and tracing | |  |  |
|  | 2-3 Construction of Regional Monitoring and Early Warning Information Management System | |  |  |
|  | 2-4 Cooperative Mechanism for Referral of Infectious Disease Patients in Areas | |  |  |
| What additional content do you think should be added or supplemented? Please rate their importance. | | | | |
| Ⅲ ability building | 3-1 Regional Emergency Response Team Building | |  |  |
|  | 3-2 Joint Emergency Training | |  |  |
|  | 3-3 Joint Emergency Drill | |  |  |
| What additional content do you think should be added or supplemented? Please rate their importance. | | | | |
| IV Information sharing | 4-1 Joint Risk Assessment and Emergency Command | |  |  |
|  | 4-2 Public and Media Information Release | |  |  |
| What additional content do you think should be added or supplemented? Please rate their importance. | | | | |
| V Public Health Governance | 5-1 Community Health Governance and Emergency Services | |  |  |
|  | 5-2 Public Health Collaborative Governance Entity Platform | |  |  |
|  | 5-3 Water Quality (Upper Reaches of Yangtze River),Air,Infectious Disease Information Sharing and Abnormal Response Mechanism | |  |  |
|  | 5-4 Participation of Socialized Organizations | |  |  |
| What additional content do you think should be added or supplemented? Please rate their importance. | | | | |
| V Talent Training and Scientific and Technological Innovation | | 6-1 Research Project Collaboration |  |  |
|  |  | 6-2 Regional School-Local Joint Construction and Sharing |  |  |
|  |  | 6-3 Construction of Cross-regional Expert Database |  |  |
| What additional content do you think should be added or supplemented? Please rate their importance. | | | | |

**Part Two**

**Experts Profile**

1. Your gender: ① Male ② Female

2. Your age: years old

3. Your educational background: ①Associate degree or below ②Bachelors degree ③Masters degree ④Doctoral degree

4. Your professional title: ①Highest-grade Senior Professional Title ②Associate Senior Professional Title ③ Intermediate Professional Title ④Junior Professional Title

5. Your professional years: years

6. Your occupation (academic/management; please fill in both if applicable):

If you are in academic domain,your research focus is and your academic position is

If you are in the management field,your primary area of focus is,and your position is

7.Your familiarity with the consultation content (mark "√" in the corresponding box)

| degree of familiarity | Very familiar | Familiar | General | Unfamiliar | Very unfamiliar |
| --- | --- | --- | --- | --- | --- |
| Expert Self-Assessment |  |  |  |  |  |

8.Your basis and degree of judgment (Please mark "√" in the corresponding box)

| judgment basis | degree of influence on judgment | | |
| --- | --- | --- | --- |
|  | big | centre | small |
| theoretical analysis |  |  |  |
| hands-on background |  |  |  |
| References |  |  |  |
| intuitive choice |  |  |  |
