# Supplementary material for: Analyzing the collaborative development needs of grassroots centers for disease control and prevention using the Kano model: A case study of China’s Chengdu–Chongqing Economic Circle
Source: PLoS One. 2026 Apr 20;21(4):e0347594. doi: 10.1371/journal.pone.0347594 (PMC13094998; doi:10.1371/journal.pone.0347594)
Supplement: S1 File — (ZIP) [file pone.0347594.s001.zip › Supporting Information - Data/Questionnaire of Collaborative Development Needs.docx]

**Survey on the Needs of Disease Control and Prevention Collaborative Development in Chengdu-Chongqing Economic Circle**

Dear Sir or Madam:

Thank you for your participation.This study aims to investigate the current status and needs of collaborative development of centers for disease control and prevention(CDCs) in the Chengdu-Chongqing Economic Circle.We kindly request your honest responses from various perspectives.Your personal information will be strictly confidential.We appreciate your understanding and participation.

1. **Basic Information**

1.Your gender ( )

A.Male B.Female

2.Your location ( )

A.Sichuan Province B.Chongqing City

3.Your field of work ( )

A.Department staff B.Administrative and Logistics Management Personnel

4.Your job title ( )

A.Middle and central management B.common worker

5.Your age ( )

A.≤30 years old B.31-50 years old C.≥51 years of age

6.What is your education level? ( )

A.Undergraduate and below B.Masters degree or above

**Ⅱ. Current Status and Attitudes of Collaborative Development of P****rimary Disease Control in the Chengdu-Chongqing Economic Circle**

1.Have you participated in any meetings or training sessions on the coordinated development of disease control within the Chengdu-Chongqing Economic Circle? ( )

A.Yes B.No

2.Have you participated in the practical work jointly carried out by the Chengdu-Chongqing Economic Circle Centers for Disease Control and Prevention (CDC)? ( )

A.Yes B.No

3.What are your expectations for the achievements of coordinated development in disease control within the Chengdu-Chongqing Economic Circle? ( )

A.High expectations B.Expect a larger

C.general expectation D.Expectations are lower

E.No expectation at all

4.Are you familiar with the policies and progress related to the coordinated development of disease control in the Chengdu-Chongqing Economic Circle? ( )

A.Very familiar B.Relatively familiar

C.General knowledge D.dont know much

E.No understanding at all

5.Are you willing to participate in the collaborative development of disease control within the Chengdu-Chongqing Economic Circle? ( )

A.Very willing B.More willing C.Generally willing

D.Not very willing E.under protest

6.Do you believe that the coordinated development of disease control in the Chengdu-Chongqing Economic Circle can promote the development of public health in both regions? ( )

A.Certainly. B.Most of the time C.once in a while

D.It probably won't work. E.Totally impossible

**III. K ANO Requirement Analysis**

**This questionnaire is based on the KANO model and aims to investigate your attitude toward the coordinated development needs of disease control in the Chengdu-Chongqing Economic Circle. Each question is presented in both positive and negative directions. Please carefully consider your stance on each item and select the option you agree with.**

Option description:

I really like it:Having this content will give you a pleasant surprise.

It should be so:You think it should be like this.

Not important: It means you don't care much,whether it's included or not.

Tolerate:Indicates that you can accept something even if you don't like it.

Dislike:You will feel very dissatisfied.

| **requirement** | **hypothesis** | Like | Should be | Does not matter | Can accept | Dislike |
| --- | --- | --- | --- | --- | --- | --- |
| Joint Construction of Party Building Brands | Yes |  |  |  |  |  |
|  | No |  |  |  |  |  |
| Jointly build and share business archives (emergency response plans,technical solutions) | Yes |  |  |  |  |  |
|  | No |  |  |  |  |  |
| Public Health Emergency Response Mechanism  (Emergency Material Reserves and Cross-regional Material Transfers) | Yes |  |  |  |  |  |
|  | No |  |  |  |  |  |
| Social Media Convergence Information Collaborative Release and Interaction | Yes |  |  |  |  |  |
|  | No |  |  |  |  |  |
| Regional school and local co-construction and sharing | Yes |  |  |  |  |  |
|  | No |  |  |  |  |  |
| Construction of cross-regional expert database | Yes |  |  |  |  |  |
|  | No |  |  |  |  |  |
| Community Health Governance and Emergency Services | Yes |  |  |  |  |  |
|  | No |  |  |  |  |  |
| Construction of Regional Monitoring and Early Warning Information Management System | Yes |  |  |  |  |  |
|  | No |  |  |  |  |  |
| Regional Collaborative Mechanism for Patient Referral in Infectious Disease | Yes |  |  |  |  |  |
|  | No |  |  |  |  |  |
| joint risk assessment and emergency command | Yes |  |  |  |  |  |
|  | No |  |  |  |  |  |
| Cross-regional case surveillance, investigation, and tracing | Yes |  |  |  |  |  |
|  | No |  |  |  |  |  |
| Performance-based pay(two options) | Yes |  |  |  |  |  |
|  | No |  |  |  |  |  |
| Public Health Collaborative Governance Entity Platform | Yes |  |  |  |  |  |
|  | No |  |  |  |  |  |
| Talent Exchange and Scientific Research Innovation Cooperation | Yes |  |  |  |  |  |
|  | No |  |  |  |  |  |
