# Supplementary material for: Analyzing the collaborative development needs of grassroots centers for disease control and prevention using the Kano model: A case study of China’s Chengdu–Chongqing Economic Circle
Source: PLoS One. 2026 Apr 20;21(4):e0347594. doi: 10.1371/journal.pone.0347594 (PMC13094998; doi:10.1371/journal.pone.0347594)
Supplement: S1 File — (ZIP) [file pone.0347594.s001.zip › Supporting Information - Data/The determination logic and formulas for the attributes of the Kano model.doc]

=IF(AND(H2=1,I2=1),"Q",IF(AND(H2=1,I2=2),"A",IF(AND(H2=1,I2=3),"A",IF(AND(H2=1,I2=4),"A",IF(AND(H2=1,I2=5),"O",IF(AND(H2=2,I2=1),"R",IF(AND(H2=2,I2=5),"M",IF(AND(H2=3,I2=1),"R",IF(AND(H2=3,I2=5),"M",IF(AND(H2=4,I2=1),"R",IF(AND(H2=4,I2=5),"M",IF(AND(H2=5,I2=1),"R",IF(AND(H2=5,I2=2),"R",IF(AND(H2=5,I2=3),"R",IF(AND(H2=5,I2=4),"R",IF(AND(H2=5,I2=5),"Q","I"))))))))))))))))
