# Supplementary material for: Analyzing the collaborative development needs of grassroots centers for disease control and prevention using the Kano model: A case study of China’s Chengdu–Chongqing Economic Circle
Source: PLoS One. 2026 Apr 20;21(4):e0347594. doi: 10.1371/journal.pone.0347594 (PMC13094998; doi:10.1371/journal.pone.0347594)
Supplement: Attachment — Submitted filename: Response to Editor and Reviewers.doc [file pone.0347594.s002.doc]

**Response to** **Editor and Reviewers**

Dear Editor and Reviewers:

Thank you for taking time out of your busy schedule to review the manuscript.

When I saw so many practical opinions, I was deeply moved. Currently, If there are any revisions that are not perfect, we will definitely improve them again as soon as possible.

The revisioninstructions are as follows:

**To Editor**

1.Editor: In the ethics statement in the Methods, you have specified that verbal consent was obtained. Please provide additional details regarding how this consent was documented and witnessed, and state whether this was approved by the IRB.

**Reply:** Additional details regarding verbal consent are described in detail in the Ethical Considerations section of the manuscript, and it is noted that this study was approved by the Ethics Committee of Shapingba District Center for Disease Control and Prevention.

2.Reviewer: Thank you for stating the following financial disclosure:

“This study was financially supported by the Chongqing Science and Health Joint Medical Research Project (2024MSXM172), and Shapingba District Science and Health Joint Medical Research Project (2024SQKWLHMS032), and Shapingba District Science and Health Joint Medical Research Project (2025SQKWLHMS072).”

Please state what role the funders took in the study.  If the funders had no role, please state: "The funders had no role in study design, data collection and analysis, decision to publish, or preparation of the manuscript."

If this statement is not correct you must amend it as needed.

Please include this amended Role of Funder statement in your cover letter; we will change the online submission form on your behalf.

**Reply:** The roles played by the three projects in this study are described in detail in the cover letter. Specifically, the three grants provided financial support for this study, enabling us to cover the labor costs of experts participating in the Delphi survey and the fees associated with manuscript publication. We have included the project information in the cover letter.

3. Reviewer: Please note that funding information should not appear in any section or other areas of your manuscript. We will only publish funding information present in the Funding Statement section of the online submission form. Please remove any funding-related text from the manuscript.

**Reply:** Thank you for your reminder. We have removed the funding information from the manuscript and added it to the cover letter.

4.Editor: In the online submission form, you indicated that “The data that support the findings of this study are available from the authors but restrictions apply to the availability of these data, which were used under license from the experts involved in the Delphi survey and the staff of CDCs who participated in the questionnaire survey for the current study, and so are not publicly available. However, data are available from the authors upon reasonable request and with permission from these experts and respondents.”

All PLOS journals now require all data underlying the findings described in their manuscript to be freely available to other researchers, either 1. In a public repository, 2. Within the manuscript itself, or 3. Uploaded as supplementary information.

This policy applies to all data except where public deposition would breach compliance with the protocol approved by your research ethics board. If your data cannot be made publicly available for ethical or legal reasons (e.g., public availability would compromise patient privacy), please explain your reasons on resubmission and your exemption request will be escalated for approval.

**Reply:** We have submitted the dataset during the revision process.

5.Editor: Your ethics statement should only appear in the Methods section of your manuscript. If your ethics statement is written in any section besides the Methods, please move it to the Methods section and delete it from any other section. Please ensure that your ethics statement is included in your manuscript, as the ethics statement entered into the online submission form will not be published alongside your manuscript.

**Reply:** Thank you for your reminder. The ethical statement appears solely in the "Methods" section of the manuscript.

6.Editor: Please remove your figures from within your manuscript file, leaving only the individual TIFF/EPS image files, uploaded separately. These will be automatically included in the reviewers’ PDF.

**Reply:** We have removed the figures from the manuscript and uploaded separate TIFF/EPS image files.

7. Editor: If tables are embedded in the manuscript and ALSO loaded as separate files, please delete the separate files, leaving only the tables in the manuscript file.

**Reply:** We have removed the separate table files and retained only the tables in the manuscript.

8.Editor: We note that the data set contains text or data that is not in English. Please note that PLOS is an English-language publisher, so we require data sets to be provided in English as well. Please upload an English-language version of your data set.

This will also allow us to determine if your data follows PLOS standards per our Data Availability policy here: https://journals.plos.org/plosone/s/data-availability.

**Reply:** All of our datasets have been uploaded in English format.

9.Editor: If the reviewer comments include a recommendation to cite specific previously published works, please review and evaluate these publications to determine whether they are relevant and should be cited. There is no requirement to cite these works unless the editor has indicated otherwise.

**Reply:** The reviewers did not suggest specific reference documents. However, in response to the reviewers' comments, we have further revised the Discussion section and attached the reference list.

**To Reviewer 1**

1.Reviewer: The description of the Delphi research method is too simplistic. It is suggested to include the process of expert selection and indicator construction.
**Reply:** Thanks for your thoughtful comment. We have provided a detailed re-description of the process of literature search and the Delphi survey. In the literature search, we re-reported the sources of the needs as well as their details. In the Delphi process, we added the sources and detailed backgrounds of the experts. At the same time, we also re-described the changes of the original need system in the two rounds of the survey, and re-drew the flowchart to facilitate a better understanding.

Figure 1. Flowchart of needs screening

Figure 2. Delphi survey

From December 15, 2024 to December 31, 2024, a literature search was conducted in databases including CNKI, Wanfang, and PubMed. The search employed the keywords “CDC,” “high-quality development,” “coordinative development,” and “integrated development” to retrieve Chinese and English literature. An indicator system for the coordinative development needs of grassroots CDCs was initially drafted, consisting of 6 primary needs and 19 secondary needs (Figure 1).

From January 13, 2025 to February 3, 2025, a Delphi survey was organized. Experts were selected based on the research objectives and principles of representativeness and authority. The selection criteria included: ① at least 5 years of experience in public health, health service management, social medicine, preventive medicine, or related fields; ② a bachelor’s degree or above; ③ an associate senior title (associate professor/associate chief physician) or higher. Through random sampling, 13 experts were recruited from the Public Health Management Professional Committee of the Sichuan and Chongqing Preventive Medicine Association. Among these experts, five held associate senior titles (41.67%) and seven held senior titles (58.33%). Their fields of expertise included public health and preventive medicine (n = 9; 75.00%), health service management (n = 2; 16.67%), and social medicine (n = 1; 8.33%). Regarding institutional affiliation, five worked in disease prevention and control centers (41.67%), one in a community health service center (8.33%), four in medical colleges or universities (33.33%), and two in tertiary hospitals (16.67%). Professional experience varied, with two experts having 10–15 years of experience (16.67%), three having 16–20 years (25.00%), and seven having over 20 years (58.33%).

During the first round of the survey, 10 experts (76.92%) provided revision suggestions; consequently, three primary needs were merged, seven secondary needs were deleted, and two secondary needs were added. In the second round, four experts (30.77%) suggested revisions that led to the merging of two secondary needs. The finalized indicator system for the coordinative development needs of grassroots CDCs comprised 4 primary needs and 13 secondary needs. The expert authority coefficient (Cr) reached 0.90 in the first round and 0.92 in the second, both exceeding the 0.70 threshold for high authority. Kendall’s coefficient of concordance for expert opinions was 0.449 and 0.452; both values were statistically significant, indicating a high degree of consensus among experts regarding the survey results (Figure 2, Table 1).

2.Reviewer: Kano sampled four cities. Please provide a representative description of the sampling cities.
**Reply:** Following your suggestion, we have provided more detailed descriptions of the sample cities.

The CCEC encompasses 15 cities in Sichuan Province and 27 districts/counties in Chongqing Municipality. Based on economic development and regional location, the CCEC comprises four areas: the Chengdu Metropolitan Area, the Chongqing Metropolitan Area, the Southern Sichuan and Western Chongqing Urban Agglomeration, and the Central Chengdu–Chongqing Urban Agglomeration. To ensure representativeness and scientificity, this study adopted a multistage sampling method to identify research subjects. In the first stage (February 13, 2025), a completely random sampling method was used to select one city from each of the four areas, resulting in two sample cities from Sichuan Province and two from Chongqing Municipality. In the second stage (February 17, 2025 to March 3, 2025), the directors of the CDCs in these four cities randomly selected staff members to serve as survey subjects.

3.Reviewer: The description mentioned as "multistage sample" merely indicates the sequence of sampling surveys conducted by the institution leaders and other personnel.

**Reply:** Thank you very much for your candid advice. Due to the unclear description, your misunderstanding occurred. The multi-stage sampling method used in this study was carried out in two stages. The first stage involved randomly selecting cities, and the second stage involved the selection of respondents by the institution leaders. We have re-described the multi-stage sampling process to make it easier to understand.

4.Reviewer: The analysis does not reflect the differences in results among different subgroups, resulting in insufficient depth of the results.
**Reply:** Thank you very much for your forward-looking suggestions. Based on your advice, we conducted a subgroup analysis of the data and further described the possible causes of the differences in the discussion section of the manuscript.

Result：Subgroup analysis reveals variations in the Kano attribute classification for specific needs; however, needs that maintain consistent attribute classifications across all subgroups are omitted from Table 6. The results indicate that respondents from Sichuan Province prioritize joint risk assessment and emergency command more significantly (*P* = 0.019). Furthermore, personnel engaged in health technology emphasize the public health emergency rescue mechanism (*P* = 0.003). Within the job position groupings, department heads and center leaders demonstrate a higher priority for mechanisms concerning the coordinated transfer and diversion diagnosis and treatment of patients with infectious diseases within the region, as well as the construction of a cross-regional expert database (*P* < 0.05).

Table 6. Subgroup analysis

| Demographic characteristics | Needs  number | Subgroup | Kano  attribute | SI | DSI | $\chi^{2}$ | P |
| --- | --- | --- | --- | --- | --- | --- | --- |
| Region | Q8 | Sichuan | M | 0.396 | −0.625 | 5.624 | 0.019 |
|  |  | Chongqing | 0 | 0.719 | −0.842 |  |  |
| Field of work | Q3 | Health technology | M | 0.364 | −0.671 | 9.321 | 0.003 |
|  |  | Administrative logistics | O | 0.765 | −0.823 |  |  |
| Job position | Q7 | Department head or center leaders | O | 0.767 | −0.433 | 5.978 | 0.016 |
|  |  | Ordinary workers | A | 0.587 | −0.333 |  |  |
|  | Q13 | Department head or center leaders | O | 0.625 | −0.719 | 4.780 | 0.031 |
|  |  | Ordinary workers | I | 0.411 | −0.438 |  |  |

Discussion:Different subgroups of the respondents have varying perceptions of specific collaborative development needs, providing a basis for the precise allocation of public health resources and the implementation of differentiated strategies. Respondents from Sichuan Province demonstrated higher concern for joint risk assessment and emergency command, which likely results from the relatively weak foundation and talent resources of grassroots CDCs in Sichuan [22]; thus, they have a more urgent need for unified cross-regional emergency risk assessment standards and collaborative emergency command. By contrast, Chongqing has initially established a digital application system for infectious disease prevention and control, and the municipal government system under direct jurisdiction has relatively strong capabilities in coordinating health resources [23]. These results suggest that the coordinated development of disease control institutions within the CCEC should consider regional differences, strengthen the standardization of joint risk assessment indicators and the collaborative mechanism of emergency command, and achieve complementary regional advantages.

Respondents engaged in health technology demonstrated greater concern for the public health emergency rescue mechanism. Health technology personnel likely possess a greater awareness of the decisive role that cross-regional material coordination capabilities play in improving emergency handling efficiency compared to those in administrative roles. Therefore, establishing a public health emergency rescue mechanism requires incorporating the opinions of health technology personnel and optimizing the standards for emergency material reserves and cross-regional allocation processes. Department heads and center leaders demonstrated higher concern for the “mechanism for the diversion and treatment of infectious disease patients within the region” and the “construction of a cross-regional expert database.” As decision-makers, these individuals can predict the potential medical resource congestion caused by excessive patient concentration and the constraints on prevention and control efficiency due to technical shortcomings [24]. Ordinary workers, whose daily work mainly involves chronic disease management and public health monitoring, rely more on local talent and have a weaker perception of the need for cross-regional expert support. At the same time, they may fear that relying on external experts might expose their own technical shortcomings, thereby reducing their expectations for this need. However, ignoring the construction of a cross-regional expert database may hide significant prevention and control risks. During the West African monkeypox epidemic[25], the public health institutions in Guinea were unable to conduct virus genotyping due to the lack of cross-regional expert support, leading to misjudgment of the transmission chain and delaying international aid [26]. This indicates that even if ordinary staff have a low awareness of this demand, building a multidisciplinary cross-regional expert database remains a vital forward-looking measure to enhance the resilience of public health emergency response [27].

5. Reviewer: "Two permits" is a policy term. It is recommended to provide a definition of the term at the first mention and not to explain it until the discussion section.
   **Reply:** Thanks for your thoughtful comment. When the "two permits" policy was first introduced, an explanation was given for this policy-related term.

Performance-based compensation. (1) Permit for medical and health institutions to exceed the current salary control levels for public institutions; (2) Permit for medical service income to be used after deducting costs and extracting various funds, and mainly for personnel rewards)

6.Reviewer: In terms of English expression: Simplify sentences, unify terminology, and complete the references in accordance with the journal requirements.
**Reply:** Thank you for your understanding. After completing the revision of the manuscript, we contacted a professional agency to polish the English expression of the manuscript, hoping to achieve the desired result.

**To Reviewer 2**

1.Reviewer: This study focuses on the collaborative development needs of grassroots Centers for Disease Control and Prevention (CDCs) in the Chengdu-Chongqing Economic Circle, innovatively applying the Kano Model to the analysis of regional collaborative needs in the public health field. The research topic aligns with global public health governance priorities and holds significant practical value and theoretical reference significance. Overall, the paper has a complete structure and clear logic with a relatively standardized research design. However, there is room for improvement in format details, language expression, methodological refinement, and discussion expansion. Detailed comments on specific dimensions are as follows:
**Reply:** All members of the research team would like to express their deep gratitude to you for your endorsement of this study. We also sincerely thank you for your valuable suggestions. We have revised the formatting details, language expression, method improvements and discussion sections of the manuscript, hoping to receive your approval.

2. Reviewer: Layout issues in tables: The content layout of "Table 5. Importance analysis scale" is chaotic, with unclear correspondence between column headers and data, affecting readability.
   **Reply:** We are very sorry for the misunderstanding caused by our handling of the three-line table. Following your suggestion, we have reorganized the layout of Table 5 to ensure that the column headings correspond one-to-one with the data.

Table5. Importance analysis scale

| Needs  number | Kano  attribute | Satisfaction  sensitivity (s) | Satisfaction sensitivity  ranking | Priority ranking  within attributes |
| --- | --- | --- | --- | --- |
| Q1 | M | 0.883 | 4 | 1 |
| Q11 | M | 0.824 | 6 | 2 |
| Q3 | M | 0.817 | 7 | 3 |
| Q5 | M | 0.775 | 9 | 4 |
| Q8 | O | 0.937 | 1 | 1 |
| Q2 | O | 0.909 | 3 | 2 |
| Q12 | O | 0.832 | 5 | 3 |
| Q6 | A | 0.922 | 2 | 1 |
| Q9 | A | 0.804 | 8 | 2 |
| Q7 | A | 0.734 | 10 | 3 |
| Q13 | I | 0.708 | 11 | 1 |
| Q10 | I | 0.469 | 12 | 2 |
| Q4 | I | 0.415 | 13 | 3 |

3.Reviewer: Insufficient clarity in the hierarchy of some section headings: For example, the subheadings in the "Methods" section do not strictly follow the "Level 1 Heading - Level 2 Heading" hierarchical logic (e.g., "Literature Review and Delphi Expert Consultation Method" and "Questionnaire Design" should be peer subheadings with inconsistent formats).
**Reply:** Thank you for your suggestion. We have revised the format of the title according to the template of PLOS again.

4.Reviewer: Grammatical and syntactic issues: Some sentences have subject-verb disagreement, tense confusion, and other errors (e.g., in "the capacity for public health governance varies significantly among CDCs across different countries and regions, with grassroots CDCs facing particular disadvantages", "facing" should be changed to "face" to maintain syntactic consistency). Some long sentences are logically redundant; for example, "Establishing stable and efficient collaborative development mechanisms among CDCs across diverse regions to maximize overall effectiveness and ensure sustainable development has emerged as a critical public health science issue" can be simplified for readability improvement.

- Inconsistent terminology use: For example, "social financial media information" appears multiple times in the text with an inaccurate expression; it should be "social integrated media information" (consistent with the secondary need expression in the table), resulting in contradictions in terminology.

- Non-idiomatic expressions: Some phrases do not conform to common academic English usage. For example, "provide scientific reference and theoretical basis" can be optimized to "provide scientific references and a theoretical foundation", and "strengthen public health systems" can be adjusted to "strengthen the construction of public health systems" to better fit the academic English context.

**Reply:** Thank you very much for your patient reading and for pointing out many places where the English expressions were not standard, which will help improve the quality of the manuscript. After completing the revision of the manuscript, we contacted a professional agency to polish the English expression of the manuscript, hoping to achieve the desired result.

5.Reviewer: High alignment between research methods and objectives: To address the core goal of "classifying and prioritizing collaborative development needs of grassroots CDCs", a combined method of "literature review + Delphi expert consultation + Kano Model" is adopted. The literature review and Delphi method ensure the scientific of the demand indicator system (expert authority coefficient Cr > 0.7, significant Kendall's coefficient of concordance), while the Kano Model's two-dimensional attribute classification advantage compensates for the defect of traditional demand analysis tools (e.g., AHP) that ignore the "negative impacts of unmet needs", enabling comprehensive capture of the different attribute characteristics of needs. Moreover, Delphi method was not clearly and explicitely descripted in the methodolgy section.

**Reply:** Thank you very much for your approval of the research concept and design. In the Delphi process, we added the sources and detailed backgrounds of the experts. At the same time, we also re-described the changes of the original need system in the two rounds of the survey, and re-drew the flowchart to facilitate a better understanding.

Figure 1. Flowchart of needs screening

Figure 2. Delphi survey

From December 15, 2024 to December 31, 2024, a literature search was conducted in databases including CNKI, Wanfang, and PubMed. The search employed the keywords “CDC,” “high-quality development,” “coordinative development,” and “integrated development” to retrieve Chinese and English literature. An indicator system for the coordinative development needs of grassroots CDCs was initially drafted, consisting of 6 primary needs and 19 secondary needs (Figure 1).

From January 13, 2025 to February 3, 2025, a Delphi survey was organized. Experts were selected based on the research objectives and principles of representativeness and authority. The selection criteria included: ① at least 5 years of experience in public health, health service management, social medicine, preventive medicine, or related fields; ② a bachelor’s degree or above; ③ an associate senior title (associate professor/associate chief physician) or higher. Through random sampling, 13 experts were recruited from the Public Health Management Professional Committee of the Sichuan and Chongqing Preventive Medicine Association. Among these experts, five held associate senior titles (41.67%) and seven held senior titles (58.33%). Their fields of expertise included public health and preventive medicine (n = 9; 75.00%), health service management (n = 2; 16.67%), and social medicine (n = 1; 8.33%). Regarding institutional affiliation, five worked in disease prevention and control centers (41.67%), one in a community health service center (8.33%), four in medical colleges or universities (33.33%), and two in tertiary hospitals (16.67%). Professional experience varied, with two experts having 10–15 years of experience (16.67%), three having 16–20 years (25.00%), and seven having over 20 years (58.33%).

During the first round of the survey, 10 experts (76.92%) provided revision suggestions; consequently, three primary needs were merged, seven secondary needs were deleted, and two secondary needs were added. In the second round, four experts (30.77%) suggested revisions that led to the merging of two secondary needs. The finalized indicator system for the coordinative development needs of grassroots CDCs comprised 4 primary needs and 13 secondary needs. The expert authority coefficient (Cr) reached 0.90 in the first round and 0.92 in the second, both exceeding the 0.70 threshold for high authority. Kendall’s coefficient of concordance for expert opinions was 0.449 and 0.452; both values were statistically significant, indicating a high degree of consensus among experts regarding the survey results (Figure 2, Table 1).

6.Reviewer: Insufficient detailed explanation of the Delphi expert consultation method: The specific selection criteria for experts (e.g., professional title, research field, years of experience) are not clearly defined; only "13 experts were selected from the Public Health Management Special Committee of the Sichuan and Chongqing Preventive Medicine Associations" is mentioned, lacking detailed descriptions of expert backgrounds, which affects the credibility of the methodology.

**Reply:** Thank you very much for such targeted advice. We refined the selection criteria for experts in the revised manuscript. At the same time, we provided detailed information about the backgrounds of the experts.

From January 13, 2025 to February 3, 2025, a Delphi survey was organized. Experts were selected based on the research objectives and principles of representativeness and authority. The selection criteria included: ① at least 5 years of experience in public health, health service management, social medicine, preventive medicine, or related fields; ② a bachelor’s degree or above; ③ an associate senior title (associate professor/associate chief physician) or higher. Through random sampling, 13 experts were recruited from the Public Health Management Professional Committee of the Sichuan and Chongqing Preventive Medicine Association. Among these experts, five held associate senior titles (41.67%) and seven held senior titles (58.33%). Their fields of expertise included public health and preventive medicine (n = 9; 75.00%), health service management (n = 2; 16.67%), and social medicine (n = 1; 8.33%). Regarding institutional affiliation, five worked in disease prevention and control centers (41.67%), one in a community health service center (8.33%), four in medical colleges or universities (33.33%), and two in tertiary hospitals (16.67%). Professional experience varied, with two experts having 10–15 years of experience (16.67%), three having 16–20 years (25.00%), and seven having over 20 years (58.33%).

7.Reviewer: The design logic of the Kano Model questionnaire can be further supplemented: The specific assignment rules for each option (e.g., "like it very much", "should be") in the "five-point Likert scale" and the operational process of attribute classification based on positive and negative answers are not explained, which is not conducive to readers replicating and verifying the research results.

**Reply:** Thank you for your suggestions that would enhance the replicability of this research.Following your suggestion, we have re-described the design logic and matching combinations of the Kano model to facilitate readers in replicating and verifying the research results.

Kano model questionnaire: This questionnaire sets positive and negative questions for each need. Each question employs a Likert five-point scale, offering five response options: “Like” (1), “Should be” (2), “Does not matter” (3), “Can accept” (4), and “Dislike” (5). By synthesizing the response combinations from the positive and negative questions, each need is categorized according to the Kano two-dimensional attribute classification matrix (Table 2). The attributes include the must-be attribute (M), one-dimensional attribute (O), attractive attribute (A), indifferent attribute (I), and reverse attribute (R). For the must-be attribute, which represents fundamental requirements, satisfaction does not significantly increase when these functions are met, but it drops sharply when they are not. By contrast, satisfaction for the one-dimensional attribute increases proportionally as features are provided and decreases when they are omitted. For the attractive attribute, satisfaction does not decrease if features are absent, but it increases substantially when they are provided. Regarding the indifferent attribute, user satisfaction remains largely unchanged regardless of whether these features are provided. Consequently, the priority order for addressing these needs follows the hierarchy of M > O > A > I. Results from the pre-survey demonstrate high reliability and validity for the instrument, with Cronbach’s α = 0.901, KMO = 0.943, and Bartlett’s test of sphericity *P* < 0.05.

Table 2. Kano model’s two-dimensional attribute classification matrix table

| Positive questions | Negative questions | | | | |
| --- | --- | --- | --- | --- | --- |
|  | Like | Should be | Does not matter | Can accept | Dislike |
| Like | Q | A | A | A | O |
| Should be | R | I | I | I | M |
| Does not matter | R | I | I | I | M |
| Can accept | R | I | I | I | M |
| Dislike | R | R | R | R | Q |

8.Reviewer: Insufficient demonstration of sample representativeness: It is not analyzed whether the distribution of the sample in demographic characteristics such as "work field" and "education level" is consistent with the overall situation of grassroots CDCs in the Chengdu-Chongqing Economic Circle, nor is the existence of sampling bias and corresponding countermeasures explained.

**Reply:**Thank you for your well-thought-out suggestions. Following your advice, we conducted subgroup analyses of the sample based on demographic characteristics information to explore the differences in the perception of demand attributes among the respondents.

Meanwhile, we provided detailed explanations regarding the existence of sampling errors in the limitation section, and proposed countermeasures and suggestions for conducting similar studies in the future.

This study has several limitations. First, the respondents were solely from the Sichuan and Chongqing regions, which may lead to selection bias. Therefore, these research results only serve as a preliminary reference for the formulation of collaborative public health policies within the CCEC and cannot be directly used as a decision-making basis for governments at all levels. Future research should include cross-regional comparative studies with areas such as the Yangtze and Pearl River Deltas to analyze commonalities and differences in grassroots CDC needs across different economic levels, thereby providing a more comprehensive reference for the construction of the national public health system. Second, this study utilized only the Kano model to conduct a quantitative assessment of potential needs. Although this method clarifies attribute classifications, it fails to reveal the underlying mechanisms that form these needs. Subsequent research should incorporate qualitative methods, such as semistructured interviews and field investigations, to explore the deep-seated factors influencing the emergence of different needs.

9.Reviewer: Overly brief interpretation of results: Only data rankings are listed, without preliminary explanation of the internal logic of some key results. For example, "talent exchange and scientific research innovation cooperation" appears in both must-be attributes and one-dimensional attributes, but the reasons for this phenomenon (e.g., differences in perceptions of the need among different respondent groups) are not explained.
**Reply:** **Thank you for your careful review of the manuscript. Here we provide one explanation and one supplementary result.**

**Explanation: The traditional Kano model determines the need attributes based on the attribute with the highest frequency, relying on the majority's opinions. This method may mask the true needs of some minority groups. The Better-Worse coefficient method, on the other hand, quantifies the impact of needs on satisfaction, avoiding subjective errors. The technical differences between the two methods may lead to differences in the classification of need attributes. Table 4 describes the frequency of different demand attributes. However, we calculated Table 4 and ultimately adopted the analysis result based on the Better-Worse coefficient method (Figure 2). Therefore, it may have caused misunderstandings among experts.**

**Modification: Based on the key information that different respondent groups have different views on the demands, we conducted an analysis of subgroups by demographics and explained the reasons why some demands were classified as different attributes.**

10.Reviewer: Unclear presentation of table data: In "Table 4. Frequency table of collaborative development needs attributes based on the Kano Model", the column alignment of some data (e.g., Q3, Q5) is chaotic, and the proportional values of attributes such as A, I, and M are not clearly corresponding.

**Reply:** **Thank you for your careful review of the manuscript. During the data analysis process, we described the frequency of different attribute occurrences by retaining two decimal places. Therefore, the total frequency values of some attributes were 1.01 or 0.99. During the revision process, we re-defined the proportion values of different attributes according to your suggestions, so that experts and readers could read them more easily.**

Table 4 Frequency table of collaborative development needs attributes based on the Kano model

| Needs  number | A | I | M | O | Q | R | SI | DSI |
| --- | --- | --- | --- | --- | --- | --- | --- | --- |
| Q1 | 10.91% | 7.27% | 54.55% | 22.72% | 1.82% | 2.73% | 0.352 | −0.810 |
| Q2 | 9.09% | 13.64% | 34.54% | 38.18% | 3.64% | 0.91% | 0.495 | −0.762 |
| Q3 | 18.18% | 10.91% | 43.63% | 22.73% | 2.73% | 1.82% | 0.429 | −0.695 |
| Q4 | 4.55% | 61.82% | 5.45% | 22.72% | 4.55% | 0.91% | 0.288 | −0.298 |
| Q5 | 16.36% | 13.64% | 47.27% | 18.18% | 3.64% | 0.91% | 0.362 | −0.686 |
| Q6 | 32.73% | 8.18% | 18.18% | 36.36% | 2.73% | 1.82% | 0.724 | −0.571 |
| Q7 | 38.18% | 22.73% | 11.82% | 22.72% | 2.73% | 1.82% | 0.638 | −0.362 |
| Q8 | 10.91% | 13.64% | 27.27% | 43.63% | 1.82% | 2.73% | 0.571 | −0.743 |
| Q9 | 31.82% | 18.18% | 15.45% | 30.00% | 2.73% | 1.82% | 0.648 | −0.476 |
| Q10 | 7.27% | 52.73% | 12.73% | 20.91% | 3.63% | 2.73% | 0.298 | −0.362 |
| Q11 | 16.36% | 13.64% | 38.18% | 27.27% | 2.73% | 1.82% | 0.457 | −0.686 |
| Q12 | 25.45% | 9.09% | 35.45% | 25.46% | 2.73% | 1.82% | 0.533 | −0.638 |
| Q13 | 13.64% | 31.82% | 18.17% | 31.82% | 2.73% | 1.82% | 0.476 | −0.524 |
| Average value | | | | | | | 0.482 | −0.586 |

11.Reviewer: Lack of statistical significance analysis of results: For example, whether there are differences in the perception of need attributes among respondents from different regions (Sichuan vs. Chongqing) and different positions (ordinary staff vs. leaders) has not been analyzed through inter-group comparison, resulting in insufficient richness of results.

**Reply:** Thank you for your targeted suggestions. Following your advice, we conducted subgroup analyses of the sample based on demographic characteristics information to explore the differences in the perception of demand attributes among the respondents.

Result：Subgroup analysis reveals variations in the Kano attribute classification for specific needs; however, needs that maintain consistent attribute classifications across all subgroups are omitted from Table 6. The results indicate that respondents from Sichuan Province prioritize joint risk assessment and emergency command more significantly (P = 0.019). Furthermore, personnel engaged in health technology emphasize the public health emergency rescue mechanism (P = 0.003). Within the job position groupings, department heads and center leaders demonstrate a higher priority for mechanisms concerning the coordinated transfer and diversion diagnosis and treatment of patients with infectious diseases within the region, as well as the construction of a cross-regional expert database (*P* < 0.05).

Table 6. Subgroup analysis

| Demographic characteristics | Needs  number | Subgroup | Kano  attribute | SI | DSI | $\chi^{2}$ | P |
| --- | --- | --- | --- | --- | --- | --- | --- |
| Region | Q8 | Sichuan | M | 0.396 | −0.625 | 5.624 | 0.019 |
|  |  | Chongqing | 0 | 0.719 | −0.842 |  |  |
| Field of work | Q3 | Health technology | M | 0.364 | −0.671 | 9.321 | 0.003 |
|  |  | Administrative logistics | O | 0.765 | −0.823 |  |  |
| Job position | Q7 | Department head or center leaders | O | 0.767 | −0.433 | 5.978 | 0.016 |
|  |  | Ordinary workers | A | 0.587 | −0.333 |  |  |
|  | Q13 | Department head or center leaders | O | 0.625 | −0.719 | 4.780 | 0.031 |
|  |  | Ordinary workers | I | 0.411 | −0.438 |  |  |

**Discussion:Different subgroups of the respondents have varying perceptions of specific collaborative development needs, providing a basis for the precise allocation of public health resources and the implementation of differentiated strategies. Respondents from Sichuan Province demonstrated higher concern for joint risk assessment and emergency command, which likely results from the relatively weak foundation and talent resources of grassroots CDCs in Sichuan [22]; thus, they have a more urgent need for unified cross-regional emergency risk assessment standards and collaborative emergency command. By contrast, Chongqing has initially established a digital application system for infectious disease prevention and control, and the municipal government system under direct jurisdiction has relatively strong capabilities in coordinating health resources [23]. These results suggest that the coordinated development of disease control institutions within the CCEC should consider regional differences, strengthen the standardization of joint risk assessment indicators and the collaborative mechanism of emergency command, and achieve complementary regional advantages.**

**Respondents engaged in health technology demonstrated greater concern for the public health emergency rescue mechanism. Health technology personnel likely possess a greater awareness of the decisive role that cross-regional material coordination capabilities play in improving emergency handling efficiency compared to those in administrative roles. Therefore, establishing a public health emergency rescue mechanism requires incorporating the opinions of health technology personnel and optimizing the standards for emergency material reserves and cross-regional allocation processes. Department heads and center leaders demonstrated higher concern for the “mechanism for the diversion and treatment of infectious disease patients within the region” and the “construction of a cross-regional expert database.” As decision-makers, these individuals can predict the potential medical resource congestion caused by excessive patient concentration and the constraints on prevention and control efficiency due to technical shortcomings [24]. Ordinary workers, whose daily work mainly involves chronic disease management and public health monitoring, rely more on local talent and have a weaker perception of the need for cross-regional expert support. At the same time, they may fear that relying on external experts might expose their own technical shortcomings, thereby reducing their expectations for this need. However, ignoring the construction of a cross-regional expert database may hide significant prevention and control risks. During the West African monkeypox epidemic[25], the public health institutions in Guinea were unable to conduct virus genotyping due to the lack of cross-regional expert support, leading to misjudgment of the transmission chain and delaying international aid [26]. This indicates that even if ordinary staff have a low awareness of this demand, building a multidisciplinary cross-regional expert database remains a vital forward-looking measure to enhance the resilience of public health emergency response [27].**

12.Reviewer: Conclusion: The discussion section focuses on core results with a certain depth, but there are problems such as a narrow discussion scope, insufficiently specific practical guidance significance, and inadequate dialogue with existing research.

**Reply:** **We made modifications to the discussion section in three aspects.**

**First, we compared the differences in the needs of different subgroups and analyzed the possible causes and proposed suggestions. Second, we conducted a horizontal comparison with existing studies. Third, based on the attributes of the needs and in combination with the actual situation of grassroots CDCs in the Chengdu-Chongqing Economic Circle, we proposed suggestions for coordinated development.**

**You can see the detailed content in the previous and subsequent responses.**

13.Reviewer: Insufficient dialogue with existing research: Only some relevant literature is cited for corroboration, without systematically sorting out the research status in the field, nor clearly defining the differences and innovations between this study and existing research (e.g., methodological differences from studies on the collaborative development of grassroots CDCs in other regions, reasons for differences in results).

**Reply:** **Thank you for your suggestion. Following your suggestions, we have further refined the manuscript.**

**In the introduction section, we have elaborated on the current research deficiencies in this field. Meanwhile, by comparing the characteristics of the kano model, the analytic hierarchy process, and the weighted method, we have expounded on the advantages and innovations of thekano model in assessing the importance of demands.**

**In the discussion section, in addition to comparing public health governance measures worldwide, we have also compared them with the demands in the Yangtze River Delta region of China and explored the possible causes.**

**For example,** permitting CDCs to conduct compensated technical services and other activities based on their responsibilities to improve salary and benefits represents the top priority for collaborative needs. This finding reflects the robust appeal among grassroots CDCs within the CCEC for enhanced compensation. Conversely, CDCs in eastern China benefit from more comprehensive fiscal guarantee systems, allowing them to prioritize digital construction and high-end technological cooperation [12], which creates a significant regional disparity compared to the CCEC.

14. Reviewer: Insufficiently specific practical guidance significance: The proposed suggestions are relatively macro (e.g., "establish a coordinated supplementary funding mechanism", "build a regional monitoring and early warning information management system"), without providing operable implementation paths combined with the localized characteristics of the Chengdu-Chongqing Economic Circle such as the administrative system and resource endowments (e.g., the source of funds for the funding mechanism, the construction subject of the information system, and data sharing standards).

**Reply:** Thank you very much for your targeted suggestions. We have combined the economic and social development level of our region and put forward more complete and practical suggestions for the coordinated development of grassroots CDCs.

Some of the suggestions are as follows.

1. Consequently, formulating the "Compensated Technical Service Management Measures" under the leadership of the governments of the two regions and in collaboration with the local Finance Bureaus, with reference to the local medical service price catalog to establish charging mechanisms for health management and public health monitoring, is of great significance for ensuring the stability of the grassroots disease control workforce.
2. Therefore, the establishment of a collaborative development alliance for CDCs and the implementation of a trans-regional material allocation mechanism—"application by primary CDCs, dual-center review, and green-channel transportation"—with the support of emergency management authorities can effectively enhance the capacity of CDCs in remote areas to respond to public health emergencies.
3. Therefore, formulating the "Specifications for Core Fields of Case Information Sharing" to clarify basic case information, diagnosis and treatment information, the number of close contacts, activity trajectories, and other relevant content is crucial for further optimizing the trans-regional case monitoring and early warning mechanism.
4. Consequently, the government-led establishment of the CCEC grassroots disease control collaborative prevention and control joint meeting—implementing monthly regular consultations and temporary consultations for sudden public health events to establish an integrated emergency command platform—has significant practical significance for breaking administrative regional barriers.

15.Reviewer: Insufficiently in-depth discussion of indifferent attributes: Only the surface reasons why needs such as "construction of a cross-regional expert database" are classified as indifferent attributes are explained, without further analyzing how to transform such "potential needs" into "effective needs" through policy guidance and mechanism innovation, which limits the practical value of the research.

**Reply:**

We would like to express our gratitude for your suggestions. In the revised manuscript, we further clarify that indifferent attributes are not unimportant, and propose corresponding measures to transform these indifferent attributes into effective tools for public health governance

Construction of a Cross-Regional Expert Database;

Establishment of Public Health Collaborative Governance Entities;

Collaborative Release and Interaction of Social Integrated Media Information.

16.Reviewer: - Failure to respond to the impact of research limitations on results: For example, the limitation of sample size prevents in-depth subgroup analysis, and it is not discussed whether this limitation will affect the reliability of demand ranking, nor are specific directions for future research proposed.

**Reply:** After reviewing all the revision suggestions from the two reviewers, we carefully examined the entire manuscript and identified the shortcomings of this study as well as proposed the future research directions. This is to provide experimental design references for other research teams to conduct similar studies in the future.

This study has several limitations. First, the respondents were solely from the Sichuan and Chongqing regions, which may lead to selection bias. Therefore, these research results only serve as a preliminary reference for the formulation of collaborative public health policies within the CCEC and cannot be directly used as a decision-making basis for governments at all levels. Future research should include cross-regional comparative studies with areas such as the Yangtze and Pearl River Deltas to analyze commonalities and differences in grassroots CDC needs across different economic levels, thereby providing a more comprehensive reference for the construction of the national public health system. Second, this study utilized only the Kano model to conduct a quantitative assessment of potential needs. Although this method clarifies attribute classifications, it fails to reveal the underlying mechanisms that form these needs. Subsequent research should incorporate qualitative methods, such as semistructured interviews and field investigations, to explore the deep-seated factors influencing the emergence of different needs.
